# Easy surgical explantation technique for sutureless Perceval S prosthesis, ‘lasso technique’: a case report

**DOI:** 10.1186/s13019-023-02160-1

**Published:** 2023-02-07

**Authors:** Yun Seok Kim, Jae Suk Yoo

**Affiliations:** 1 Department of Thoracic and Cardiovascular Surgery, Keimyung University Dongsan Hospital, Keimyung University College of Medicine, Daegu, Republic of Korea; 2 Department of Thoracic and Cardiovascular Surgery, Asan Medical Center, University of Ulsan College of Medicine, 88 Olympic-ro 43-Gil, Songpa-Gu, Seoul, Republic of Korea

**Keywords:** Sutureless aortic valve replacement, Prosthetic valve failure, Prosthetic valve explantation

## Abstract

**Background:**

Due to structural valve deterioration of sutureless aortic prosthesis, there is a need for explantation of the prothesis. We introduce a surgical technique to explant sutureless aortic prosthesis, which has a self-expanding stent incorporated into the aortic wall.

**Case presentation:**

An 82-year-old man who had undergone sutureless aortic valve replacement 6 years previously underwent redo-aortic and mitral valve replacement because of severe prosthetic aortic valve stenosis and mitral regurgitation. The sutureless prosthesis was explanted using ‘lasso technique’. The patient was discharged after 7 days without complications.

**Conclusions:**

We presented a useful technique to explant a sutureless aortic prosthesis.

**Supplementary Information:**

The online version contains supplementary material available at 10.1186/s13019-023-02160-1.

## Background

The Perceval S sutureless prosthetic aortic valve has a self-expanding nitinol alloy stent instead of a sewing cuff and shows favorable hemodynamic performance [1]. Despite the higher incidence of permanent pacemaker implantation, it shows similar rates of 30-day mortality and significant paravalvular leakage compared with conventional bioprosthetic aortic valves [2]. Additionally, the sutureless prosthetic aortic valve is suitable for minimally invasive approaches, such as right anterior thoracotomy or partial sternotomy. Despite these advantages, these leaflets are made of bovine pericardium, and structural valve deterioration is inevitable. Therefore, the need for explantation is gradually increasing, and surgeons should understand the innate properties of sutureless prostheses.

## Case presentation

An 82-year-old man was referred to our department for surgery because of severe prosthetic aortic valve stenosis and mitral regurgitation. He had undergone sutureless aortic valve replacement (Perceval S Medium, LivaNova, London, UK) 6 years previously. He also had chronic kidney disease requiring hemodialysis, diabetes, hypertension, and gastric submucosal tumor.

Transthoracic echocardiography revealed a mean aortic transvalvular pressure gradient of 68 mmHg and a thickened, retracted anterior mitral leaflet, causing severe regurgitation. The left ventricular ejection fraction was 66%. Our multidisciplinary heart team decided to explant the prosthetic aortic valve and perform surgical aortic and mitral valve replacements.

Before median sternotomy, the right common femoral artery and vein, and the right internal jugular vein were cannulated, and cardiopulmonary bypass was instituted. After aortic cross-clamping, antegrade cold crystalloid cardioplegia (modified del Nido solution) was administered. We performed an extended transseptal atriotomy followed by oblique aortotomy. We used the ‘lasso technique’ to create a secure dissection plane between the intima and the sutureless aortic prosthesis, which has a self-expanding stent incorporated into the aortic wall. The procedure details were described in our previous report [3]. In summary, a 2–0 polyester purse-string suture was passed along the edge of the frame and secured using a tourniquet to collapse the prosthesis, creating a secure dissection plane. Dissection was then carried out using the tourniquet as a handle to control the prosthesis (Additional file 1: Video 1). Previously [3], we used two tourniquets to explant a transcatheter aortic valve prosthesis but in the present case, only one tourniquet was sufficient. Adhesions were more severe at the annulus than aortic wall. During debridement of the residual calcification at the aortic annulus, we noticed some intimal denudation, and we repaired it with an autologous pericardial patch using 5–0 polypropylene running suture.

The native mitral valve was excised and a 25 mm Hancock II (Medtronic, Minneapolis, MN, USA) prosthetic mitral valve was implanted using 13 everting buttress sutures of 2–0 polyester with pledgets. Subsequently, an Avalus (Medtronic) 19 mm prosthetic aortic valve was implanted using 12 non-everting sutures of 2–0 polyester with pledgets. All sutures were tied using Cor-Knot (LSI Solutions, NY, USA) automated titanium fasteners. Cardiopulmonary bypass was weaned off smoothly. Cardiopulmonary bypass and aorta cross-clamping times were 146 and 108 min, respectively. Postoperative transthoracic echocardiography showed no aortic regurgitation and a mean transvalvular pressure gradient of 12 mm Hg, with normal ventricular function. However, indexed effective orifice area of the prosthetic aortic valve was 0.64 cm^2^/m^2^ indicating severe patient-prosthesis mismatch. The mechanical ventilation time was 6 h, and the patient was discharged after 7 days without complications.

This study was approved by the Institutional Review Board (IRB) and ethics committee of the Asan Medical Center (approval number, 2022–1096; approval date, 10 August 2022). The requirement for informed consent was waived because of the retrospective nature of the study design. All relevant data are included in the manuscript and its supporting files.

## Discussion

Unlike conventional bioprosthetic aortic valves, sutureless prostheses have self-expanding properties and are incorporated into the aortic wall, annulus, and left ventricular outflow tract, and caution is needed during explantation. The self-expanding property of the sutureless prosthesis is similar to that of the transcatheter aortic valve replacement (TAVR) prosthesis, and there have been several reports on explantation of TAVR prosthesis [3, 4]. While TAVR is performed without excision of the calcified aortic valve and annulus, sutureless AVR is performed after excision, and the self-expanding stent is more severely incorporated into the surrounding tissue. In the present case, the outflow ring of the sutureless prosthesis was easily freed from the aorta. However, it is not always easy and lavage of the nitinol frame with cold saline could help shrink the frame and aid easier removal. Comparing at the aortic wall, adhesions were more severe at the annulus and an unintended injury occurred. We repaired it with a patch of bovine pericardium and multiple pledgeted sutures which made the annulus narrow causing patient-prosthesis mismatch eventually. Considering large BSA (1.77 m^2^) of the patient, 19 mm prosthetic valve was expected to cause patient-prosthesis mismatch. However, given old age of the patient and surgical risk, we did not proceed any annular widening procedures.

## Conclusion

We used the ‘lasso technique’ to create a dissection plane between the self-expanding prosthesis and intima, as we previously reported [3]. As shown by the successful outcomes of this case, we believe that the ‘lasso technique’ is still useful for explanting sutureless prostheses.

## Supplementary Information

**Additional file 1: Video 1. **The ‘lasso technique.’ A 2-0 polyester purse-string suture was passed along the edge of the frame and fastened using a tourniquet to collapse the prosthesis making a secure dissection plane. Then, dissection was carried out using the tourniquet as a handle controlling the prosthesis.

## Data Availability

All relevant data are included in the manuscript and its supporting files.
